# Santa Claus' Garner

**Published:** 1886-12-25

**Authors:** 


### Santa Claus' Garner.

By our Special Commissioner.

A Children's Paradise.

That ancient home of dancing and dining and general revelry, whose utter worldliness is only partially redeemed by occasional meetings—generally in May—of the unco' guid—I refer to Willis's Rooms, was the rendezvous of many hundreds of folk, big and little, last Saturday and Monday. The door had been opened for little more than an hour when I paid my visit on Saturday morning, and already the great silver salver, of which a stalwart *commissionnaire* had charge, was piled high with visiting-cards—the production of which was the only passport required. In the room, where pleasure-seekers and gourmets ordinarily leave their coats and hats, or where, in the middle of a bright summer day, bishops and missionaries hold forth with varying degrees of zeal and eloquence, the visitors were confronted with a spectacle which made the elders rub their eyes with astonishment, and the children open theirs with delight. What is it? A glorified Lowther Arcade, arranged with a taste and splendour which would make the proprietors of the stalls in that well-known bazaar green with envy? An incarnation of all the fairy tales from Cinderella to the Brothers Grimm and Hans Andersen? A medley more wondrously varied than even Alice could have imagined after her voyage to Wonderland? It is all this and more. I have given it its proper name at the head of this article. It is Santa Claus' Garner; and the harvestmen who have been working with such magnificent success are the proprietor of *Truth* and the gentlemen who assist him in the conduct of that Journal. They may well be proud of the result of their labours, and of the skill and taste which they have displayed in arranging the riches which they have collected for that most genial of Christmas sprites. The history of the *Truth* toy competition and distribution, which was commenced on a modest scale six years ago, was narrated in a recent number of The Hospital, and it is only for me to try and describe a few, a very few, of the toys gathered together last Saturday and Monday at Willis's Rooms.

Fifteen thousand Toys.

But where shall I begin? There are fifteen thousand or so of toys of all sorts, from the tiniest doll to the most rampant and roaring lions ever seen out of an African jungle. At one end of the room the pile of playthings reaches up to the ceiling, and but a spare inch of uncovered board is to be seen either on the stalls which line the walls, or on the two large stands which monopolise the centre of the room, and make locomotion, even at this early hour in the morning, exceedingly difficult. Mr. Horace Vowles, whose master mind has conceived and planned all the details of this display, says the arrangement of the room was the work of one day only, to which one can only respond in the words of the immortal Dominie, "Prodigious!" One of Mr. Vowles' assistants pathetically complained that the affair is getting too big, although at the same time there was an undercurrent of pride in his tones. Indeed, it is evident that all the gentlemen engaged must have their hearts in the work; merely paid assistance would not have arranged this room with less than three days' labour.

The Purse of Fortunatus.

But now for the toys themselves. And first, I will look at the table against the end wall, where, as I have said, the exhibits are piled up to the ceiling. In the centre of the stand is a glass case containing 9,000 bright new sixpences—the anonymous gift which, like the *Truth* toy fund itself, gets bigger and bigger every Christmas. Speculations as to who can be the owner of this Fortunatus' purse, which year after year is shaken with ever-increasing vigour into the basket of Santa Claus' husbandmen, are of course rife. But *Truth*, in this instance at least, keeps her own counsel, and we go away no wiser than we came. The case of silver is guarded on either side by a fierce-looking lion, which emits a most natural roar when a string underneath the mane is pulled.

Something like a Show.

All down the table there are other animals,—wild and tame sheep, tigers, dogs, wolves, donkeys, cows, and so on—and all of a very decent size, mind you. The secret of their voice was well preserved in every case; in some you pulled the tail, in others you bowed the head, in others again you turned the head aside; and, as each child who passed deemed it a duty to test the articulatory powers of the toy, the room was filled with sound resembling a farm-yard and a zoological garden combined. Amongst the row of animals was a white pig. The children pushed and pulled and squeezed him, but not a squeak could be got. The poor animal was in great danger of being mauled to death, when some kind friend affixed a notice: "Please, do not touch. This is *not* a mechanical toy." Piggy was the silent member of the herd! On this stall, too were some hundreds of dolls, supplied from the *Truth* fund, and beautifully dressed by friends of the Kilburn Sisters at 4½*d.* each. A cheap dressmaker's bill, isn't it? Above here again were piled, one above another, toys of every description—perambulators, Noah's Arks, clowns, harlequins, locomotives, horses, cats and dogs,—whilst great masks grinned and gaped a welcome to all who passed underneath.

Nor are the old people forgotten in this Christmas Saturnalia which the readers of *Truth* provide for the London hospitals and workhouses. At one end of the table was piled 74 lbs. of tobacco and $27\frac{3}{4}$ lbs. of snuff, and at the other 222 lbs. of tea; whilst in opposite corners of the room stood 2,000 pots of jam, sent by Messrs. Clarke, Nicholls and Coombe, and 14,000 crackers, the gift of Messrs. Tom Smith and Co.

The "Old Folks" remembered.

Then what shall I say of the two other long stalls of bought toys? Imagine, dear readers, all the best things of all the London toyshops heaped together, and you will have some idea of the *tout ensemble*. There were scores of the sailor dolls about one of which *Truth* told so pathetic a story last week. There were wicker-work locomotives with their tenders stuffed full of blondes and brunettes all beautiful, all dressed in holiday attire. There was a musical-box of the American pattern, the tunes of which were stamped on rolls of paper, and ground out with a handle (I forget the fancy name the makers had given to it). There were drums and tops for the noisy children, and dominoes and picture-books for the quiet ones, and hundreds of little bags filled with sweets for little ones, whose eyes will glisten with delight when the gifts are distributed at the bed-sides this Christmas morning.

What Happiness!

I must not forget, too, some surprise Jam-pots, labelled thus, "Home-made Real Jam. This jam is guaranteed to be made of the fresh fruit, and with refined sugar only." You touch a spring, the cover falls back, and up jumps a certain grand old man, with smiling face, and large collar. I wondered whether it was a Liberal or a Tory who had made this curious presentation? There were two or three rocking-chairs, too, which some children's wards, I know of, will covet. They are of basket-work with seats for three little children, one at each end, and one perched high up in the middle. What an endless source of delight these will be to many a little convalescent!

Real Jam!

But it is time to turn to the home-made toys which are displayed on two large tables in the middle of the room. I think these are more interesting than any, for a gift upon which many a busy hour has been spent speaks more of the kindness of the donor than the most gorgeous present bought in a shop. The competition this year is for fancy-dressed dolls, and a number of clever people of all ages and conditions have joined in the friendly rivalry. The prizes are not awarded until after the show has closed, so I am not able to say upon whose shoulders the honours have descended. It goes without saying that there are any number of beauties in patches and powder, of gallants in ruffles and embroidered coats, Hamlets and Ophelias, Patiences and Grosvenors, Spanish ladies and Moorish gentlemen—not one of the characters of a fancy ball are wanting. Two natives of Malay, as gorgeous as yellow satin and embroidery can make them, are conspicuous among the motley group. And one little maiden sends a lovely blonde in swansdown, with a letter of explanation that "it was made last summer, before it was announced that the feature of the Exhibition would be fancy-dressed dolls." She signs herself "Bootle's Baby." A charming brunette, dressed in white, is sent by the fond papa of the little lady who dressed it. Missy writes from school to her "dear dad," and signs herself "Your loving Babs"—and a very loving Babs she must be to take so much trouble over her Christmas present to the poor children of the London hospitals and workhouses.

Home-made Toys.

And now a word about the home-made models, which are of great variety and number. But stay! I had forgotten to mention a magnificent bridal procession of about a dozen dolls sent by Madame Louise, which has the place of honour at the central table. There are not so many dolls' houses among the models as one might suppose. The workers have too much exercised their ingenuity for that; but here are Kindergarten schools, country fairs, the story of Red Riding Hood and the Wolf, a water picnic, a fancy fair (this last a very wonder of minute work), and fairy tales all worked in miniature, with every detail as complete as if the boys and girls who made them had been doing nothing else but make models since they could u e their hands. To go through them all would occupy far greater space than THE HOSPITAL– with all its enthusiasm for the matter in hand—could spare me. I will only say that I left Willis's Rooms full of gratitude to the thoughtful people who have started and maintained the *Truth* toy-fund; and to the hundreds of people, old and young, rich and poor, who have done so much to brighten the lives of the deserted and sick children of London at this holy season of "peace and good will towards men."

Full of Gratitude.

